# Canadian national surveys on pandemic influenza preparations: pre-pandemic and peri-pandemic findings

**DOI:** 10.1186/1471-2458-13-271

**Published:** 2013-03-25

**Authors:** Paul Ritvo, Daniel F Perez, Kumanan Wilson, Jennifer L Gibson, Crissa L Guglietti, C Shawn Tracy, Cecile M Bensimon, Ross EG Upshur

**Affiliations:** 1 School of Kinesiology and Health Science, York University, 4700 Keele Street, Toronto, M3J 1P3, Canada; 2 Research, Prevention and Cancer Control, Cancer Care Ontario, 505 University Avenue, Toronto, M5G2L7, Canada; 3 University of Toronto Joint Centre for Bioethics, 55 College, Street, Suite 754, Toronto, M5T 1P8, Canada; 4 Department of Medicine, Ottawa Health Research Institute, University of Ottawa, 725 Parkdale Ave, Ottawa, K1Y 4E9, Canada; 5 Primary Care Research Unit, Sunnybrook Health Sciences Centre, 2075 Bayview Avenue, Toronto, M4N 3M5, Canada; 6 Dalla Lana School of Public Health, 155 College St., Toronto, On, M5T 3M7; 7 Department of Family and Community Medicine, University of Toronto, 263 McCaul Street, Toronto, M5T 1W7, Canada; 8 Institute of Health Policy, Management and Evaluation, University of Toronto, 155 College Street, Toronto, M5T 1P8, Canada

**Keywords:** Pandemic influenza, Canada, Survey research, Public health ethics, Bioethics, H1N1

## Abstract

**Background:**

Prior to the 2009 H1N1 Influenza pandemic, public health authorities in Canada and elsewhere prepared for the future outbreak, partly guided by an ethical framework developed within the Canadian Program of Research on Ethics in a Pandemic (CanPREP). We developed a telephone-based survey based on that framework, which was delivered across Canada in late 2008. In June, 2009, the WHO declared pandemic Phase 6 status and from the subsequent October (2009) until May 2010, the CanPREP team fielded a second (revised) survey, collecting another 1,000 opinions from Canadians during a period of pre-pandemic anticipation and peri-pandemic experience.

**Methods:**

Surveys were administered by telephone with random sampling achieved via random digit dialing. Eligible participants were adults, 18 years or older, with per province stratification approximating provincial percentages of national population. Descriptive results were tabulated and logistic regression analyses used to assess whether demographic factors were significantly associated with outcomes, and to identify divergences (between the pre-pandemic and intra-pandemic surveys).

**Results:**

N = 1,029 interviews were completed from 1,986 households, yielding a gross response rate of 52% (AAPOR Standard Definition 3). Over 90% of subjects indicated the most important goal of pandemic influenza preparations was saving lives, with 41% indicating that saving lives solely in Canada was the highest priority and 50% indicating saving lives globally was the highest priority. About 90% of respondents supported the obligation of health care workers to report to work and face influenza pandemic risks excepting those with serious health conditions which that increased risks. Strong majorities favoured stocking adequate protective antiviral dosages for all Canadians (92%) and, if effective, influenza vaccinations (95%). Over 70% agreed Canada should provide international assistance to poorer countries for pandemic preparation, even if resources for Canadians were reduced.

**Conclusions:**

Results suggest Canadians trust public health officials to make difficult decisions, providing emphasis is maintained on reciprocity and respect for individual rights. Canadians also support international obligations to help poorer countries and associated efforts to save lives outside the country, even if intra-national efforts are reduced.

## Background

In April 2009, a novel H1N1 influenza virus emerged from Mexico [1] and spread rapidly through North America and the rest of the globe [2,3]. Margaret Chan, the Director General of the World Health Organization (WHO), raised the pandemic alert to its maximum level (Level 6) [4], where it remained for fourteen succeeding months (until August 2010) [5]. While these events have largely receded from the public consciousness, we are reminded of a possible recurrence by events like the culling of 156,439 chickens in China's Xinjiang region (July, 2012) following a confirmed outbreak of the H5N1 avian flu.

Despite considerable concern about H1N1 in 2009, its impact in terms of morbidity and mortality was relatively mild, although significant societal and health care system disruptions were observed [6-8]. In October 2009 (four months after the start of the Level 6 alert) we modified a previously implemented survey and conducted a second national Canadian survey on pandemic preparedness and management ethics [9] which remained in the field until May 2010 during considerable fluctuations in risk perceptions, influenced by personal experiences and media reports on during the outbreak [10,11]. Survey responses were likely influenced by these socio-environmental fluctuations [12].

Our survey items were based on themes identified in *Stand on Guard for Thee: Ethical Considerations in Preparedness Planning for Pandemic Influenza* (SOGFT) [13], a report that significantly influenced the WHO’s global pandemic ethics consultation [14,15]. The four themes that guided this survey work were *Duty to Care* (responsibilities and rights of healthcare workers), *Restrictive Measures* (quarantine, vaccine use, school/transit closures, etc.), *Priority Setting* (priorities for distribution of limited healthcare resources in a pandemic), and *Global Governance* (roles and responsibilities of Canada and other countries to the global community generally and to poorer countries in particular). The majority of items used in Survey 1 were replicated in Survey 2 enabling comparisons of Canadian responses before and during the pandemic. We were able to compare responses from before the pandemic when the level of risk and likelihood of a pandemic were unknown, with the peri-pandemic period, when anxieties and risk perceptions fluctuated on the basis of direct experience, media, and advancing scientific inquiry. Several new items were also developed for Survey 2 which addressed additional issues relevant to ethical debates in pandemic management.

### Survey rationale

Engaging the public on ethical issues related to pandemic planning and response is a significant part of morally defensible policy-making. As previously noted in the Ritvo et al. [9] survey paper, the Eleventh Futures Forum [14] on the ethical governance of pandemic preparedness stressed public engagement in pandemic planning initiatives. Public engagement via surveys can capture immediate public responses to current issues, informing decisions on policy and shaping public health communications. The purpose of this study was to provide an up to date look at the opinions of Canadians regarding these issues, as well as to assess the stability of those opinions over time. As our first survey gathered the public’s responses to particular items prior to the WHO declaration of a pandemic, our second survey was aimed at gathering the public’s responses during the H1N1 pandemic period.

## Methods

The study authors created the original survey by developing questions adapted from the themes identified by *Stand on Guard for Thee* (SOGFT) [13] a survey format compatible with administration via random digit telephone dialing. Decisions about item construction were based on investigator consensus and small-group pilot testing. New and repeated items resulted from iterative revisions during which selection was based on item performance (observed in Survey 1 analyses) and judgments of face validity (in relation to the SOGFT framework). Some items required minor revision in relation to changing conditions (e.g., references to ‘bird/avian flu’ changed to ‘H1N1/swine flu’) while novel items were included relating to sources of health information, vaccine attitudes, preferred decision makers and trust. Items were carefully constructed at an 8th grade reading level to precisely convey content to a population varying in educational and ethnocultural backgrounds.

### Sampling

Random sampling was achieved via random digit dialing with random telephone numbers from across Canada, purchased from Sampling Modeling and Research Technologies, Inc. (SMRT, Markham, Ontario). Eligible participants included all adults over 18 years of age with proficiency in either English or French. Screening of participants was based on standardized inquiries regarding age, provincial residency and gender. To effectively represent nationwide attitudes, we stratified the sample per province to obtain subsamples equivalent to each province’s proportional contribution to the national population. The territories were not included in this survey. Participants were provided the option of the survey being administered in English or French, and all contacts with residents of Quebec were initiated in French. Survey administration was conducted by the Institute for Social Research at York University.

### Analyses

Descriptive results of the frequencies of responses to all survey questions were tabulated. Logistic regression analyses were used to identify demographic factors significantly associated with survey outcomes and reported in terms of odds ratios and 90% confidence intervals. Specific factors were treated as independent (predictor) variables and key findings as dependent (outcome) variables. These analyses addressed the following factors: gender; age; educational background; employment status; residence (urban vs. rural); marital status (married vs. unmarried) and family status (children vs. no children). The variables were dichotomized as follows: age [18–52 years vs. 53–98 years], marital status [married (including common law) vs. unmarried (single, separated, divorced, widowed)], education [<college/university vs. ≥ college/university] and employment [employed (full time and part time) vs. unemployed (including students, retired, disability)].

## Results

### Response rate

A total of 3,188 randomized telephone numbers were drawn, of which 1,986 numbers were estimated to be households. Households included all numbers where successful interviews occurred, plus all households where refusals or callbacks were noted, as well as an assumption that the majority of the "never answered” numbers were households. N=1,029 interviews were completed from 1,986 households yielding a gross response rate of 52% (AAPOR Standard Definition 3). However, at telephone numbers where an identifiably eligible respondent answered, the response rate was 88%. The greatest number of repeated calls needed to get a single completion was 46, and approximately 10% of the interviews were completed on a 14th or subsequent call attempt.

### Survey findings

Table 1 contains demographic data, with demographic results from the initial survey presented for reference. Additional file 1: Table S1 presents the results of selected questions in common between survey 1 and 2 for comparison purposes. Full survey results are found in Additional file 2: Table S2. Below is a summary which organizes survey responses by topic area and presents the results of selected questions as percentages. When demographic factors were associated with statistically significant differences in responses to questions in logistic regression, odds ratios are presented.

**Table 1:** Survey 1 & 2 demographic results

	**SURVEY 1 Mean(SD)**	**SURVEY 2 Mean(SD)**
**Age** in Years (n = 1000)	51 (17)	52(16.4)
Refusals (n=29)		
Total (n = 1029)		
	Frequency (%)	Frequency (%)
**Province**		
Newfoundland	8(1%)	18(1.7%)
Prince Edward Island	2(<1%)	6(<1%)
Nova Scotia	14(2.8%)	37(3.6%)
New Brunswick	13(2.6%)	28(2.7%)
Quebec	117(23.3%)	204(19.8%)
Ontario	197(39.3%)	406(39.5%)
Manitoba	19(3.8%)	41(4.0%)
Saskatchewan	14(2.8%)	39(3.8%)
Alberta	52(10.4%)	112(10.9%)
British Columbia	65 (13%)	138(13.4%)
Total	501	1029
**Gender**		
Male	176(35%)	425(41.3%)
Female	325(65%)	604(58.7%)
Refused	0	0
Total	501	1029
**Marital Status**		
Married or Common Law	311(62%)	611(59.4%)
Widowed	30(6%)	99(9.6%)
Separated	14(3%)	38(3.7%)
Divorced	31(6%)	75(7.3%)
Never Married	107(21%)	190(18.5%)
Refused	8(2%)	16(1.6%)
Total	501	1029
**Has Children**		
Yes	376(75%)	776(75.4%)
No	118(24%)	245(23.8%)
Refused	7(1%)	8(0.8%)
Total	501	1029
**Education**		
< High School	39(8%)	92(8.9%)
Completed High School	126(25%)	258(25.1%)
Completed some College/University	49(10%)	99(9.6%)
Completed College/University degree	232(47%)	457(44.4%)
Professional Degree	- - (–)	13(1.3%)
Masters	36(7%)	73(7.1%)
PhD	10(2%)	20(1.9%)
Refused	9(2%)	17(1.7%)
**Ethnicity**		
Caucasian	407(81%)	915(88.9%)
Asian	21(4%)	36(1.4%)
Black	21(4%)	17(1.7%)
Aboriginal	11(2%)	16(1.6%)
Latin American/Hispanic	8(2%)	8(0.8%)
Other	28(6%)	15(1.5%)
Don’t know/ Refused	5 (1%)	22(2.2%)
**Employment**		
Full-time	261(52%)	541(52.6%)
Part-time	53(10%)	113(11.0%)
Not Employed (Retired, unemployed, disability)	181(36%)	358(34.8%)
Other	6(1%)	4(0.4%)
Refused	6(1%)	13(1.3%)
**Residence**		
Urban	303(60%)	707(68.7%)
Rural	194(39%)	312(30.3%)
Refused	4(1%)	10(1.0%)
	(**Survey 2 Only**)	Frequency (%)
**Household Income** < $20,000		21(2.0%)
$20,000 - $29,000		23(2.2%)
$30,000 - $39,000		21(2.0%)
$40,000 - $49,000		18(1.7%)
$50,000 - $59,000		18(1.7%)
$60,000 - $69,000		9(0.9%)
$70,000 - $79,000		15(1.5%)
$80,000 - $89,000		9(0.9%)
$90,000 - $99,000		8(0.8%)
$100,000 - $119,000		13(1.3%)
$120,000 - $149,000		10(1.0%)
$150,000 +		10(1.0%)
Refused		854(83.0%)
Total		1029(100%)

### Pre-pandemic / peri-pandemic comparison items

#### Main purpose of pandemic plan

Responses to Survey 1 and 2 were compared, using a criterion of fluctuation greater than 10 percentage points to highlight survey items which may reflect substantial changes in public opinion. This value was selected to make the most substantial opinion shifts stand out more readily for analysis, given the large number of survey items. However, it must be noted that changes smaller than 10 percentage points could be significant, particularly for items where responses might predictably be highly homogenous under routine conditions. For example, in response to the question regarding the main purpose of a Canadian Pandemic Plan, the most frequent responses were ‘saving as many lives as possible in Canada” (36% in Survey 2 vs. 41% in Survey 1) and “saving as many lives as possible, globally” (40% in Survey 2 vs. 50% in Survey 1). While other response options to this question were somewhat more frequently endorsed in Survey 2 compared to Survey 1, they were still infrequently endorsed compared to the two options concerning saving lives.

#### Health care worker obligations, global obligations and antiviral access

As seen in Additional file 1: Table S1, fluctuations in responses to Survey 1 and 2 also did not exceed 10 percentage points in the domains referred to above, with respondents to Survey 2 nearly as supportive as respondents to Survey 1 of health care worker (HCW) obligations to report to work and face risks during patient-care under pandemic conditions (90% agreed in survey 1 vs. 83% agreed in survey 2). In contrast to Survey 1, Survey 2 respondents were more in favor of considerations for HCWs with young children or elderly relatives, and those with a serious health condition that increased risk. The Survey 2 respondents were also less decisively in favor of consequences such as loss of employment or professional license, if HCWs did not report for work (49.4% agreed vs. 45.5% disagreed in survey 2 and 48% agreed vs. 38% disagreed in survey 1). Survey 2 respondents were similarly less supportive of additional disability and death benefits at no charge for health care workers during a flu crisis but more supportive of government rights to conscript health care workers during a pandemic (52% agreed with conscription in survey 2 vs. 43.7% disagreed, whereas only 47% agreed in survey 1 vs. 43% disagreed).

In terms of global obligations to help poorer countries during a pandemic, even if the result was a reduction of resources for Canadians, a majority were in favor (58.6% agreed and 38.4% disagreed) although a smaller majority than in the first survey (70% agreed and 18% disagreed in survey 1). In both surveys, an overwhelming majority favored provision of adequate amounts of antiviral medications to every Canadian (82.1% agreed in survey 2, while 92 % agreed in survey 1).

In terms of the relevant regression analyses, those over the age of 53 years (vs. younger, OR = 1.51, p < 0.01, 1.11 – 2.06) and males (vs. females, OR = 1.42, p < 0.01, 1.08-1.88) were more likely to think health care workers who don’t report to work during an H1N1 flu pandemic should face loss of employment or professional license. It is notable that this gender difference was also observed in survey 1, where females were more likely to agree that health care workers with dependents should *not* have to report to work (OR = 2.07, p < 0.05, 1.05 – 4.19). In survey 2, males were more likely to think that governments should reserve the right to conscript health care workers during an H1N1 flu pandemic (OR = 1.55, p < 0.01, 1.17-2.05) a finding that again can be compared with survey 1, where females were more likely to not agree (OR = 2.10, p < 0.05, 1.05 – 4.19) with government conscription of health care workers.

In terms of global obligations, respondents with ≥ college/university education (OR = 1.69, p < 0.001, 1.25 – 2.29) and without children (OR = 1.83, p < 0.01, 1.26 – 2.67) were more likely to think Canadians should help poorer countries even if it reduced resources for Canadians, while those who were unmarried (OR = 1.48, p < 0.05, 1.05 – 2.09), with <college/university education (OR = 2.70, p < 0.001, 1.99 – 3.67), and the unemployed (OR = 1.43, p = 0.05, 1.00 – 2.04) were all more likely to think all Canadian needs should be taken care of before sending resources to poorer countries. In survey 1, unemployed individuals (OR = 3.14, p < 0.05, 1.21 – 8.15) were more likely to disagree with provision of international assistance and those unemployed (OR = 2.72, p < 0.05, 0.16 – 4.83) and living in rural communities (OR = 3.00, p = 0.01, 1.34 – 6.74) were both more likely to disagree specifically with Canadian donation of more than 10% of total resources to poorer countries. Finally, in survey 2 those with <college/university education (OR = 1.99, p = 0.001, 1.35 – 2.93) were more likely to feel that adequate amounts of antiviral medication should be provided to every Canadian. If anti-viral medication supplies were insufficient, those unmarried (OR = 1.57, p < 0.01, 1.15-2.14), <college/university education (OR = 2.28, p < 0.001, 1.68 – 3.10), and the unemployed (OR = 1.46, p < 0.05, 1.06-2.02) were all more likely to feel there should be an equal chance (e.g., lottery) of receiving the medication as opposed to a priority-based distribution. In survey 1, participants with < college/university education were significantly less likely (OR = 2.25, p < 0.05, 1.17 – 4.35) to support government-set priorities regarding distribution of antiviral medications.

#### Access priorities to hospital treatment and antiviral medications

Once again, the groups rated as having highest priority for hospital treatment/resources remained stable between surveys (health care workers infected while serving patients, children and the sickest patients) although their relative order of priority changed in survey 2 (health care workers were given priority over children and sickest patients) compared to survey 1 (children were given priority over health care workers and the sickest patients). The differences in prioritization responses varied by less than 5 percentage points. The same pattern was evident with respect to access to antiviral drugs, with top priorities accorded to health care workers and children in both surveys. However, adults with chronic diseases were rated the third highest priority in survey 2, advancing ahead of public safety/social service workers and adults with dependents that had been rated higher in survey 1.

### Novel items

#### H1N1 impact and public facility closure

About 19.7% of respondents felt becoming infected with H1N1 would have the most impact on them, followed by confronting a dysfunctional health system (17.9%) and loss of salary (17.5%). Approximately 76.3% of respondents felt schools and daycare facilities should be closed to prevent H1N1 infections. A strong majority of respondents felt it was acceptable to close entertainment venues (76.9%), require people to work from home (85.8%), close schools and daycares (77.3%), and limit non-urgent hospital visits/services (87.3%). Nearly half of respondents (46.8%) thought it was acceptable to shut down or limit public transportation to reduce H1N1 infection. In terms of regression results, the only significant finding was that males were more likely to think that schools and daycare facilities should be closed to prevent H1N1 flu infection (OR = 2.07, p < 0.001, 1.47 – 2.91).

#### H1N1 vaccine issues

About 74% of respondents felt health care workers or other essential service workers had a duty to be immunized against H1N1, but this decreased to 56.7% if rare side effects were discovered to be associated with the vaccine. Seventy-seven percent felt that those injured by vaccine side effects should be compensated by the vaccine manufacturers, while 60% felt compensation should come from the government. Respondents stated they had the most trust in nationally selected scientific advisory boards (31.8%) and in the federal government (19.7%) to oversee a vaccine-injury compensation program. In considering a trade-off between safety testing and timely delivery of vaccines, 47.1% felt there should be no compromise in safety, while 25% felt there could be minor reductions in safety testing, and 23.6% felt there could be moderate reductions in safety testing. Altogether, 57% of respondents stated they would obtain a H1N1 vaccine during the fall months. In regression findings, those with <college/university education (OR = 1.61, p < 0.001, 1.16 – 2.23) and the unemployed (OR = 1.48, p < 0.05, 1.01 – 2.16) were both more likely to think that HCWs should be immunized against H1N1. The unemployed (OR = 1.41, p < 0.05, 1.01 – 1.96) also were more likely to think HCWs should be immunized even if rare vaccine related side effects were discovered. Those with ≥ college/university education (OR = 1.74, p = 0.001, 1.27 – 2.37) and those over 53 years of age (OR =1.74, p < 0.01, 1.14 – 2.17) were more likely to state that they would obtain an H1N1 vaccine.

#### Sources of H1N1 information and reasons for public trust and distrust

Respondents indicated they mainly accessed H1N1 information from the chief public health officer (83.4%) and their physician (94.8%), further indicating trust for information obtained from physician experts (35%) and the Public Health Agency of Canada (23.1%). Respondents also stated that receiving inaccurate information (55.7%), discovering that a specific group of Canadians were receiving less than a fair share of resources (51.4%), or that wealthier people were receiving special privileges (73.4%) were situations that would decrease respondents’ trust in public authorities by ‘a great deal’,. Trust in public authorities would be *increased* ‘a great deal’ if adequate resources were directed to those most in need (62.2%). Respondents felt accurate communications (33%) and adequate resources provided to those most in need (25.1%) would be the most important observations in increasing their trust in public authorities.

In terms of H1N1 information access, females were more likely to get information from their physician (OR = 1.35,p < 0.05, 1.01 – 1.80) while those ≥ college/university education (OR = 1.58, p = 0.001, 1.18 – 2.12), employed (OR = 1.73, p = 0.001, 1.25 – 2.40), younger (between 18–52 years) (OR = 1.52, p < 0.01, 1.12 – 2.07) and female (OR = 1.68, p < 0.001, 1.26 – 2.24) were more likely to access government websites. Additionally, those aged 53 and older (OR = 1.64, p < 0.01, 1.17 – 2.30) were more likely to access information from newspapers, while those younger than 53 were more likely to access Google news (OR = 1.41, p < 0.05, 1.01 – 1.97) and YouTube (OR = 3.26, p < 0.01, 1.48 – 7.19). Females (OR = 1.89, p < 0.05, 1.06 – 3.39), <college/university education (OR = 1.94, p < 0.05, 1.03 – 3.66), and those aged 18–52 (OR = 4.86, p < 0.001, 2.46 – 9.59) were more likely to gain information from Facebook or MySpace.

## Discussion

These results demonstrate the complexity involved in understanding the public’s view of ethical issues in pandemic planning. While there is strong agreement and stability in many domains, there are important variations in opinion across the two time points. As Survey 2 was administered during the second wave of H1N1 in Canada, it reflects perceptions *during* the pandemic as contrasted with Survey 1 responses obtained when the pandemic was anticipated (but undefined). As a result, these data represent an important vantage point for pre-pandemic and peri-pandemic comparisons.

Most opinions of respondents were relatively stable in comparison to the previous survey. Canadians believe the main goal of a pandemic response is to reduce influenza-related mortality, with reduction of global mortality an important dimension of this objective. Respondents believed the H1N1 pandemic would have a significant impact and were willing to tolerate societal disruptions to increase safety, with a majority ready to accept closures of schools and daycare facilities to prevent infections although only a minority were prepared to shut down public transit. Canadians also acknowledged the difficulties that healthcare professionals (HCPs) face in personal health decisions, while still demanding high standards of care and professional practice responsibility. For example, Canadians strongly agreed that health care workers should face risks and provide care during an H1N1 pandemic (before and during pandemic conditions), but this view was tempered by support for excusing those with health conditions that put them at greater risk, or those with domestic (dependent) care obligations. This is further balanced by a consistent favoring of HCPs in prioritizing them for health-related resources, although it is impossible to determine whether this prioritization originates from a desire to maximize health care resources in a time of need, or a desire to ensure fairness or justice for HCPs. In trust-related questions, HCPs are seen as the most trusted purveyors of health information and the ones with the highest credibility to make difficult decisions regarding intensive care access. There is, nonetheless, substantial disagreement about the acceptability of sanctions for health care workers failing to provide care during a pandemic and the acceptability of conscription as a means of ensuring sufficient health care workers are available for pandemic response.

Respondents remain supportive of public health social-distancing measures, with the exception of stopping public transit. Respondents also trust public health officials as sources of information in pandemics which may seem surprising given the confusion around messaging in the H1N1 vaccine roll-out, particularly concerning the unclear or changing information regarding vaccination priority groups during the 2009 H1N1 pandemic.

Priority setting remains a controversial area as seen in the reluctance of a significant majority to identify categories of lowest priority. Future qualitative analyses from focus group data collected in association with this project (guided by discussion questions aligned with survey items) will permit greater insight into such responses. With current data, some of the underlying meanings of responses remain ambiguous. It is possible that participants are making a clear decision to not identify low priority groups based on a considered opinion that all Canadian subgroups are of relatively high importance, such that it may not be possible for or acceptable to respondents to determine priorities that may undermine valued notions of equality and fairness. However, it is also possible that participants are simply uncomfortable identifying low priority groups to surveyors as a matter of social desirability. The two highest priority populations shifted between survey 1 and 2, but only with respect to whether health care workers or children were the first priority. Both surveys provide potentially important insights into Canadian perceptions on the obligations of health care workers, the acceptability of public health measures, priority setting, communication and public trust, and indicate a number of avenues for further exploration with implications for infectious disease response planning and the day-to-day operations of our healthcare system.

### Limitations

The strengths of this survey include the use of random digit dialing to access a large, representative Canadian sample and Time 1-Time 2 comparisons of baseline survey items. A carefully planned theoretical framework\underlies the survey structure. Nonetheless, there are limitations related to the overrepresentation of females and the large proportion of respondents self-identifying as white (in an increasingly diverse national population) and middle-aged. These demographics may better reflect the population with land-based telephone lines in Canada versus those with cellular phones; hence another limitation is the absence of a protocol for accessing cellular numbers. It is also worth noting that aside from the 2009 pandemic, other world-changing events occurred, or were occurring, between Surveys 1 & 2, including the initiation of world-wide banking and economic crises. As the data is based on a quantitative protocol, we can only hypothesize about the reasons for responses, but qualitative analyses, currently underway as another component of the overall CanPREP project, will soon supplement survey findings.

## Conclusions

Despite the understandable response fluctuations apparently related to sampling occurring during pandemic conditions, there was observable stability in the responses to survey 2 compared to the first (pre-pandemic) survey. In terms of new items, strong support was found for the duty or obligation of health care workers to be immunized against H1N1 and for vaccine manufacturers (77%) or government (60%) compensations, if immunization resulted in injurious side effects. Compensation could be overseen preferably by nationally selected scientific advisory boards (31.8%) and/or the federal government (19.7%), although opinion was divided on the question. Public trust appeared closely associated with accurate information and equitable resource-sharing. Trust in public authorities would be decreased ‘a great deal’ with discoveries that a specific group of Canadians were receiving less than a fair share of resources (51.4%), and wealthier people receiving special privileges (73.4%); it would be *increased* ‘a great deal’ if adequate resources were directed to those most in need (62.2%). In summary, surveying the public on bioethical issues related to pandemic prevention and management is important. Increasing emphasis on public consultations will allow policymakers and public health authorities to target activities towards identified priorities, using modes of communication that address the loci of trust held by the broadest proportion of the population. Such targeted consultations can allow for better responses to the particular needs of so-called ‘vulnerable’ groups such as senior citizens, First Nations, and/or low-SES populations.

## Competing interests

The authors declare that they have no competing interests.

## Authors’ contributions

PR, KW, JLG, CST, JXN, ARJ, and REGU drafted survey items. CG, PR, and REGU conducted statistical analyses. CG, PR, DP, KW, JLG, CST, JXN, ARJ, and REGU interpreted statistical analyses. PR, DP, KW, JLG, CG, CST, JXN, ARJ, and REGU drafted the manuscript. PR, KW, CST, and REGU determined methodology. PR, KW, CST, and REGU supervised telephone survey administration. All authors read and approved the final manuscript.

## Pre-publication history

The pre-publication history for this paper can be accessed here:

http://www.biomedcentral.com/1471-2458/13/271/prepub

## Supplementary Material

Additional file 1: Table S1 Comparison of Selected Responses to Identical Items in Survey 1 and Survey 2. Click here for file

Additional file 2: Table S2 Full Results per Question (Survey 2). Click here for file
