# Supplementary material for: Canadian national surveys on pandemic influenza preparations: pre-pandemic and peri-pandemic findings
Source: BMC Public Health. 2013 Mar 25;13:271. doi: 10.1186/1471-2458-13-271 (PMC3627899; doi:10.1186/1471-2458-13-271)
Supplement: Additional file 1: Table S1 — Comparison of Selected Responses to Identical Items in Survey 1 and Survey 2. [file 1471-2458-13-271-S1.doc]

Table 2 - Comparison of Selected Responses to Identical Items in Survey 1 and Survey 2

| What should be the main purpose of the Canadian Pandemic Plan? | Survey 1 (%) | Survey 2 (%) |
| --- | --- | --- |
| Saving as many lives as possible, in Canada | 41 | 36 |
| Saving as many lives as possible, globally | 50 | 40 |
| Maintain social order | 4 | 8 |
| Protect human rights | 3 | 6 |
| Prevent economic decline | 1.3 | 6 |
| Refused/Don’t know | 0.7 | 4 |

|  | **Agree** | | **Disagree** | |
| --- | --- | --- | --- | --- |
|  | **Survey 1** | **Survey 2** | **Survey 1** | **Survey 2** |
| Health care workers should report to work and face all risks when caring for patients during an H1N1 pandemic | 90 | 83.4 | 5 | 13.8 |
| Health care workers who do not come to work during an H1N1 pandemic should face loss of employment or loss of professional license | 48 | 49.4 | 38 | 45.5 |
| Health care workers who must care for their young children or elderly relatives should not be expected to work during a pandemic | 57 | 66.4 | 30 | 29.3 |
| Governments should reserve the right to conscript health care workers during an H1N1 pandemic | 47 | 52.3 | 43 | 43.7 |
| If a health care worker has a serious health condition that can increase their risk, they should not have to come to work during an H1N1 flu pandemic | 89 | 88.1 | 9 | 10.3 |
| Governments should provide additional disability insurance and death benefits at no charge for health care workers at risk during an H1N1 flu crisis | 85 | 72.2 | 9 | 24.1 |
| There should be adequate amounts of antiviral medications provided to every Canadian | 92 | 82.1 | 4 | 14.2 |
| Even if it reduces resources to Canadians, wealthy countries like Canada should still provide help to poorer countries during a pandemic | 70 | 58.6 | 18 | 38.4 |

| ***Antiviral Medication –***  If the government set priorities for who should receive antivirals, what priority would you rank: | **High Priority** | | **Moderate Priority** | | **Low Priority** | |
| --- | --- | --- | --- | --- | --- | --- |
|  | **Survey 1** | **Survey 2** | **Survey 1** | **Survey 2** | **Survey 1** | **Survey 2** |
| Children | 90 | 70.5 | 9 | 23.1 | 1 | 4.2 |
| Seniors | 48 | 39.7 | 35 | 36 | 17 | 21.9 |
| Adults with chronic illnesses | 39 | 63.7 | 31 | 25.8 | 30 | 6.7 |
| Health care workers | 95 | 88.5 | 5 | 9.8 | 0 | 0.4 |
| Public safety and social service workers | 76 | 50.1 | 23 | 40.3 | 1 | 7.3 |
| Single adults | 40 | 8.9 | 49 | 44.3 | 11 | 42.6 |
| Adults with dependents | 78 | 49.7 | 21 | 41.6 | 1 | 6.5 |
| Public officials | 32 | 12.1 | 50 | 46.5 | 18 | 38.4 |

| ***Hospital Treatment Access – If there were not enough intensive care resources, which would be the highest/lowest priority:*** | **Highest Priority** | | **Lowest Priority** | |
| --- | --- | --- | --- | --- |
|  | **Survey 1** | **Survey 2** | **Survey 1** | **Survey 2** |
| The sickest patients | 75 | 70.2 | 15 | 19.1 |
| The patients most likely to recover | 54 | 37.4 | 28 | 15.6 |
| Health care workers infected with pandemic flu while serving patients | 85 | 73.2 | 14 | 22.5 |
| Elderly | 32 | 32 | 42 | 44.4 |
| Public officials | 24 | 11.7 | 51 | 50.8 |
| Children | 88 | 72.8 | 12 | 22.4 |
| Single adults | 35 | 10.3 | 51 | 52.9 |
| Adults with dependents | 74 | 51.2 | 25 | 42.1 |
