# Supplementary material for: Canadian national surveys on pandemic influenza preparations: pre-pandemic and peri-pandemic findings
Source: BMC Public Health. 2013 Mar 25;13:271. doi: 10.1186/1471-2458-13-271 (PMC3627899; doi:10.1186/1471-2458-13-271)
Supplement: Additional file 2: Table S2 — Full Results per Question (Survey 2). [file 1471-2458-13-271-S2.doc]

**Table 3: Full Results per Question (Survey 2)**

|  | Big | Moderate | Small/No Impact | Don’t Know | Refused | N/A | Total |
| --- | --- | --- | --- | --- | --- | --- | --- |
| 3) How much of an impact would the following have on you during an H1N1 outbreak?  a) School Closures  b) Major Work Absence  c) Major H1N1 Illness  d) Major Salary Loss  e) Dysfunctional Health Systems | 201(19.5)  337(32.8)  594(57.7)  475(46.2)  623(60.5) | 172(16.7)  208(20.2)  262(25.5)  161(15.6)  254(24.7) | 604(58.7)  337(32.8)  116(11.3)  244(23.7)  124(12.10 | 11(1.1)  8(0.8)  55(5.3)  20(1.9)  27(2.60 | 3(0.3)  3(0.3)  2(0.2)  2(0.2)  1(0.1) | 38(3.7)  136(13.2)  -  127(12.3)  - | 1029  1029  1029  1029  1029 |

|  | Frequency (%) |  | Frequency (%) |
| --- | --- | --- | --- |
| 4) Which of these would have the most impact on you?  School Closing  None would have an impact  Absent from work  H1N1 illness  Losing salary  Dysfunction in health system  Don’t know  Total | 218(21.2)  48(4.7)  251(24.4)  211(20.5)  278(27.0)  22(2.1)  1(0.1)  1029 | 5) If you had to choose one purpose for the Canadian Pandemic Plan, what would it be?  Saving as many lives as possible, in Canada  Saving as many lives as possible, globally  Maintaining social order  Protecting human rights  Preventing economic decline  Don’t know  Refused  Total | 374(36.3)  412(40.0)  81(7.9)  66(6.4)  65(6.3)  30(2.9)  1(0.1)  1029 |

|  | Agree | Disagree | Don’t know | Refused | Total |
| --- | --- | --- | --- | --- | --- |
| 6) Health care workers and other essential service workers have an ethical duty to be immunized against H1N1 to protect the people that they serve | 878(85.4) | 133(12.9) | 18(1.7) | - | 1029 |
| 7) Health care workers should report to work and face all risks when caring for patients during an H1N1 pandemic | 858(83.4) | 142(13.8) | 28(2.7) | 1(0.1) | 1029 |
| 8) Health care workers who do not come to work during an H1N1 pandemic should face loss of employment or loss of professional license | 509(49.4) | 468(45.5) | 49(4.8) | 3(0.3) | 1029 |
| 9) Health care workers who must care for their young children or elderly relatives should not be expected to work during a pandemic | 683(66.4) | 301(29.3) | 43(4.2) | 2(0.2) | 1029 |
| 10) Governments should reserve the right to conscript health care workers during an H1N1 pandemic | 538(52.3) | 449(43.7) | 38(3.7) | 4(0.4) | 1029 |
| 11) If a health care worker has a serious health condition that can increase their risk, they should not have to come to work during an H1N1 flu pandemic | 907(88.1) | 106(10.3) | 15(1.5) | 1(0.1) | 1029 |
| 12) Governments should provide additional disability insurance and death benefits at no charge for health care workers at risk during an H1N1 flu crisis | 743(72.2) | 248(24.1) | 35(3.4) | 3(0.3) | 1029 |
| 13) Schools and daycare facilities should be closed if it prevents H1N1 flu infection | 779(76.3) | 215(20.9) | 26(2.5) | 2(0.2) | 1029 |

|  | Acceptable | Not Acceptable | Don’t Know | Refused | Total |
| --- | --- | --- | --- | --- | --- |
| 14) To reduce the spread of the flu, how acceptable is it:  a) to close entertainment venues  b) Require people to work at home  c) Shut down/limit public transit use  d) Close schools and day cares  e) Limit non-urgent hospital visits/services | 771(74.9)  883(85.6)  482(46.8)  795(77.3)  899(87.3) | 250(24.3)  125(12.1)  519(50.4)  219(21.3)  108(10.5) | 8(0.8)  19(1.8)  28(2.7)  15(1.5)  21(2.0) | -  2(0.2)  -  -  1(0.1) | 1029  1029  1029  1029  1029 |
|  | Agree | Disagree | Don’t Know |  | Total |
| 18) There should be adequate amounts of antiviral medications provided to every Canadian. | 844(82.1) | 146(14.2) | 39(3.8) |  | 1029 |
|  | Set Priorities | Equal Chance | Don’t know | Refused | Total |
| 19) If there were not enough antiviral medicine, which statement comes closest to what you think? | 552(53.6) | 443(43.1) | 31(3.0) | 3(0.3) | 1029 |

| 20) If the government set priorities for who should receive antivirals, what priority would you rank:  a) Children under 18  b) Older persons above 75  c) Adults w/ chronic illness  d) Health care workers  e) Public safety and social service workers  f) Single adults  g) Adults w/ dependents  h) Public officials | High  725(70.5)  409(39.7)  655(63.7)  911(88.5)  516(50.1)  92(8.9)  511(49.7)  124(12.1) | Moderate  238(23.1)  370(36.0)  265(25.8)  101(9.8)  415(40.3)  456(44.3)  428(41.6)  478(46.5) | Low  43(4.2)  225(21.9)  69(6.7)  4(0.4)  75(7.3)  438(42.6)  67(6.5)  395(38.4) | Don’t Know  21(2.0)  22(2.1)  38(3.7)  11(1.1)  21(2.0)  36(3.5)  19(1.8)  29(2.8) | Refused  2(0.2)  3(0.3)  2(0.2)  2(0.2)  2(0.2)  7(0.7)  4(0.4)  3(0.3) | Total  1029  1029  1029  1029  1029  1029  1029  1029 |
| --- | --- | --- | --- | --- | --- | --- |

|  | Frequency (%) |  | Frequency (%) |
| --- | --- | --- | --- |
| 20i) Who should have the highest priority for antiviral protection?  None have highest priority  Children  Older persons  Adults w/ chronic illness  Health care workers  Public safety and social service workers  Adults w/ dependents  Public officials  Don’t know  Refused  Total | 27(2.6)  293(28.5)  20(1.9)  92(8.9)  476(46.3)  31(3.0)  41(4.0)  2(0.2)  46(4.5)  1(0.1)  1029 | 20j) Who should have the lowest priority for antiviral protection?  None have lowest priority  Children  Older persons  Adults w/ chronic illness  Public safety and social service workers  Single adults  Adults w/ dependents  Public officials  Don’t know  Total | 355(34.5)  8(0.8)  123(12.0)  31(3.0)  14(1.4)  257(25.0)  5(0.5)  207(20.1)  29(2.8)  1029 |

| 22) If there were not enough intensive care resources, what priority would you rank: | High | Moderate | Low | Don’t Know | Refused | Total |
| --- | --- | --- | --- | --- | --- | --- |
| a) Sickest patients | 722(70.2) | 197(19.1) | 54(5.2) | 49(4.8) | 7(0.7) | 1029 |
| b) Patients most likely to recover | 385(37.4) | 450(15.6) | 161(15.6) | 27(2.6) | 6(0.6) | 1029 |
| c) Health care workers | 753(73.2) | 232(22.5) | 18(1.7) | 22(2.1) | 4(0.4) | 1029 |
| d) Public safety and social service workers | 383(37.2) | 532(51.7) | 78(7.6) | 30(2.9) | 6(0.6) | 1029 |
| e) Older persons above 75 years | 329(32.0) | 47(44.4) | 205(19.9) | 33(3.2) | 5(0.5) | 1029 |
| f) Adults with chronic illnesses | 557(54.1) | 331(32.2) | 109(10.6) | 27(2.6) | 5(0.5) | 1029 |
| g) Public officials | 120(11.7) | 523(50.8) | 33(34.3) | 29(2.8) | 4(0.4) | 1029 |
| h) Children under 18 | 749(72.8) | 230(22.4) | 25(2.4) | 21(2.0) | 4(0.4) | 1029 |
| i) Single adults | 106(10.3) | 544(52.9) | 336(32.7) | 37(3.6) | 6(0.6) | 1029 |
| j) Adults with dependents | 527(51.2) | 433(42.1) | 36(3.5) | 28(2.7) | 5(0.5) | 1029 |

|  | Frequency (%) |  | Frequency (%) |
| --- | --- | --- | --- |
| 22k) Which group should have the highest priority for intensive care resources?  None have the highest priority  Sickest patients  Patients most likely to recover  Health care workers  Public safety and social service workers  Older persons above 75 years  Adults with chronic illnesses  Public officials  Children under 18  Single adults  Adults with dependents  Don’t know  Refused  Total | 24(2.3)  126(12.2)  46(4.5)  282(27.4)  30(2.9)  22(2.1)  62(6.0)  2(0.2)  340(33.0)  4(0.4)  44(4.3)  42(4.1)  5(0.1)  1029 | 22l) Which group should have the lowest priority for intensive care resources?  None have the lowest priority  Sickest patients  Patients most likely to recover  Health care workers  Public safety and social service workers  Older persons above 75 years  Adults with chronic illnesses  Public officials  Children under 18  Single adults  Adults with dependents  Don’t know  Refused  Total | 400(38.9)  20(1.9)  82(8.0)  4(0.4)  11(1.1)  96(9.3)  40(3.9)  171(16.6)  3(0.3)  181(17.6)  2(0.2)  18(1.7)  1(0.1)  1029 |

|  | Very Acceptable | Not Acceptable | Don’t know | Refused | Total |
| --- | --- | --- | --- | --- | --- |
| 23) Who should make decisions about access to intensive care resources  a) Physicians  b) Clinicians or health workers  c) Hospital administrators  d) Government officials  e) Judges  f) Courts  g) Patients and families | 984(95.6)  901(87.6)  511(49.7)  300(29.1)  316(30.7)  347(33.8)  706(68.6) | 16(1.6)  91(8.8)  495(48.1)  699(67.9)  688(66.7)  650(63.2)  287(27.9) | 23(2.2)  33(3.2)  18(1.7)  26(2.5)  22(2.1)  28(2.7)  31(3.0) | 6(0.6)  4(0.4)  5(0.5)  4(0.4)  5(0.5)  4(0.4)  5(0.5) | 1029  1029  1029  1029  1029  1029  1029 |
|  | Agree | Disagree | Don’t know | Refused | Total |
| 24) Even if it reduces resources to Canadians, wealthy countries like Canada should still provide help to poorer countries during a pandemic | 603(58.6) | 395(38.4) | 28(2.7) | 3(0.3) | 1029 |

|  | Below 7-10% | 7-10% | Above 7-10% | Don’t know | Refused | Total |
| --- | --- | --- | --- | --- | --- | --- |
| 25) What proportion of total resources devoted to H1N1 flu preparation in Canada should be used to help poorer countries? | 220(21.4) | 588(57.1) | 161(15.6) | 55(5.3) | 5(0.5) | 1029 |

|  | Yes | No | Don’t Know | Refused | Total |
| --- | --- | --- | --- | --- | --- |
| 26) Should all Canadian needs be taken care of for dealing with an H1N1 outbreak before any resources are delivered to help poorer countries prepare for the flu? | 665(64.8) | 316(30.7) | 4(4.4) | 3(0.3) | 1029 |
| 27) Do you believe healthcare workers or other essential service workers have a duty to be immunized against H1N1 to protect the people they serve? | 756(73.5) | 244(23.7) | 27(2.6) | 2(0.2) | 1029 |
| 28) What if there are rare vaccine side-effects? | 583(56.7) | 377(36.6) | 65(6.3) | 4(0.4) | 1029 |
| 29) Should those injured by a rare but serious side-effect receive some form of compensation form the government? | 627(60) | 335(32.6) | 67(6.5) | - | 1029 |
| 30) What about compensation from vaccine manufacturers? | 800(77.7) | 184(17.9) | 43(4.2) | 2(0.2) | 1029 |

|  | Frequency (%) |
| --- | --- |
| 31) Who would you most trust to oversee a vaccine-injury compensation program?  Federal government  Provincial government  Nationally selected scientific advisory boards  Nationally selected citizen groups  Provincially selected scientific advisory boards  Provincially selected citizen group  Don’t know  Refused  Total | 203(19.7)  64(6.2)  327(31.8)  113(11.0)  92 (8.9)  106(10.3)  113(11.0)  11(1.1)  1029 |

|  | Yes | No | Don’t Know | Refused | Total |
| --- | --- | --- | --- | --- | --- |
| 32) Would you personally accept an H1N1 vaccine if it is available to you this fall? | 587(57.0) | 371(36.1) | 70(6.8) | 1(0.1) | 1029 |
| 33) Source of H1N1 information:  a) Doctor  b) Government website  c) TV news program  d) Newspapers  e) Google news  f) Facebook/MySpace  g) YouTube  h) Family and friends  i) Co-workers  j) Twitter  k) Other | 347(33.7)  423(41.1)  856(83.2)  739(71.8)  299(29.1)  78(7.6)  47(4.6)  766(74.4)  507(49.3)  21(2.0)  307(29.8) | 673(65.4)  600(58.3)  168(16.3)  285(27.7)  720(70.0)  945(91.8)  978(95.0)  258(25.1)  365(25.5)  1000(97.2)  715(69.5) | 1(0.1)  1(0.1)  -  1(0.1)  5(0.5)  2(0.2)  -  -  149(14.0)  -  - | 8(0.8)  5(0.5)  5(0.5)  4(0.4)  4(0.4)  4(0.4)  4(0.4)  5(0.5)  6(0.6)  8(0.8)  7(0.7) | 1029  1029  1029  1029  1029  1029  1029  1029  1029  1029  1029 |
| 34) For information during H1N1 emergency, who do you trust for information/directions?  a) Chief public health officer  b) Your doctor  c) Television broadcasters  d) Family and friends  e) Co-workers  f) Provincial premier  g) Prime minister  h) Newspaper journalists | 858(83.4)  976(94.8)  382(37.1)  592(57.5)  342(33.2)  407(39.6)  470(45.7)  347(33.7) | 130(12.6)  42(4.1)  586(56.9)  388(37.7)  473(46.0)  572(5.6)  509(49.5)  614(59.7) | -  4(0.4)  -  -  172(16.7)  -  -  - | 40(3.9)  7(0.7)  60(5.8)  48(4.7)  39(3.8)  49(4.8)  47(4.6)  66(6.4) | 1029  1029  1029  1029  1029  1029  1029  1029 |

|  | Frequency (%) |  |  |
| --- | --- | --- | --- |
| 35a) Who do you trust most for H1N1 information?  Public health officials  Physician expert  Nurse  World Health Organization (WHO)  Public Health Agency of Canada (PHAC)  Other  Don’t know  Refused  Total | 174(16.9)  360(35.0)  38(3.7)  169(16.4)  238(23.1)  7(0.7)  41(4.0)  2(0.2)  1029 | 35b) Who do you trust second most?  Public health officials  Physician expert  Nurse  World Health Organization (WHO)  Public Health Agency of Canada (PHAC)  No one else trusted  Don’t know  Refused  Missing  Total | 210(20.4)  269(26.1)  101(9.8)  136(13.2)  234(22.7)  6(0.6)  63(6.1)  3(0.3)  7(0.7)  1029 |
| 35c) Who do you trust third most?  Public health officials  Physician expert  Nurse  World Health Organization (WHO)  Public Health Agency of Canada (PHAC)  No one else trusted  Don’t know  Refused  Missing  Total | 212(20.6)  165(16.0)  158(15.4)  182(17.7)  205(19.9)  12(1.2)  82(8.0)  6(0.6)  7(0.7)  1029 |  | |

|  | A great deal | A little | No difference | Don’t know | Refused | Total |
| --- | --- | --- | --- | --- | --- | --- |
| 36a) How much would your trust in public authorities be reduced:  1) If receiving inaccurate information  2) If a group of Canadians was receiving less than their fair share of resources  3) If some wealthier people were getting special privileges  4) If Canada was not doing a good job helping poorer countries | 573(55.7)  529(51.4)  755(73.4)  333(32.4) | 261(25.4)  321(31.2)  136(13.2)  477(46.4) | 165(16.0)  127(12.3)  112(10.9)  181(17.6) | 28(2.7)  48(4.7)  23(2.2)  35(3.4) | 2(0.2)  4(0.4)  3(0.3)  3(0.3) | 1029  1029  1029  1029 |
| 36b) How much would your trust in public authorities be increased:  1) If receiving regular accurate information  2) If H1N1 decisions through open public engagement/consultation  3) If adequate resources were being directed to those in greatest need  4) Receiving updates about how Canada is helping poorer countries | 577(6.1)  428(41.6)  640(62.2)  368(35.8) | 294(28.6)  394(38.3)  272(26.4)  452(43.9) | 134(13.0)  155(15.1)  84(8.2)  178(17.3) | 22(2.1)  47(4.6)  29(2.8)  28(2.7) | 2(0.2)  5(0.5)  4(0.4)  3(0.3) | 1029  1029  1029  1029 |

|  | Frequency (%) |  | Frequency (%) |
| --- | --- | --- | --- |
| 36c1) Which of these is most important to increasing your trust?  None would have greatest increase  Accurate communications  Public engagement and consultation  Adequate resources directed to those in greatest need  Updates on what Canada is doing to help internationally  Don’t know  Refused  Total | 220(21.4)  340(33.0)  90(8.7)  258(25.1)  91(8.8)  29(2.8)  1(0.1)  1029 | 36c2) Second most important  No second most important  Accurate communications  Public engagement and consultation  Adequate resources directed to those in greatest need  Updates on what Canada is doing to help internationally  Don’t know  Refused  Total | 659(64.0)  73(7.1)  94(9.1)  165(16.0)  34(3.3)  4(0.4)  -  1029 |
| 36c3) Third most important  No third most important  Accurate communications  Public engagement and consultation  Adequate resources directed to those in greatest need  Updates on what Canada is doing to help internationally  Don’t know  Refused  Total | 860(83.6)  14(1.4)  61(5.9)  51(5.0)  39(3.8)  4(0.4)  -  1029 |  | |

|  | No compromise | Minor reductions in safety testing is acceptable | Moderate reductions is safety testing is acceptable | Don’t know | Refused | Total |
| --- | --- | --- | --- | --- | --- | --- |
| 37) Given the pressure to deliver the H1N1 flu vaccine on time for the regular flu season, how willing are you to trade safety testing for on-time vaccine delivery? | 485(47.1) | 258(25.1) | 243(23.6) | 35(3.4) | 8(0.8) | 1029 |
